# A novel haemoplasma species identified in archived primate blood smears

**DOI:** 10.1016/j.vetmic.2010.11.016

**Published:** 2011-05-05

**Authors:** Emily N. Barker, Chris. R. Helps, Harold Neimark, Iain R. Peters, Wallace Peters, Séverine Tasker

**Affiliations:** a School of Veterinary Sciences, University of Bristol, Langford, Bristol, BS40 5DU, United Kingdom; b Department of Microbiology & Immunology, College of Medicine, State University of New York, Brooklyn, NY 11203, USA; c Department of Infectious and Tropical Diseases, London School of Hygiene and Tropical Diseases, London, WC1E 7HT United Kingdom

**Keywords:** Haemoplasma, Quantitative polymerase chain reaction, Primate, *Aotus trivirgatus*, RNase P RNA gene, Phylogeny

## Abstract

In order to confirm a microscopic diagnosis of ‘eperythrozoonosis’ made over 40 years ago in a captive owl monkey (*Aotus trivirgatus*), DNA was extracted from archived fixed and stained blood smears and subjected to generic haemotropic mycoplasma (haemoplasma) quantitative real-time PCR (qPCR) and a human glyceraldehyde-3-phosphate dehydrogenase qPCR as an amplification control. The qPCRs confirmed the extraction of host DNA from the samples and the presence of a haemoplasma species. Partial 16S rRNA and ribonuclease P ribosomal gene fragments were amplified by PCR, cloned and sequenced. Sequence data and phylogeny showed the owl monkey haemoplasma to lie in the haemominutum clade of haemoplasmas, most closely related to ‘*Candidatus* Mycoplasma kahaneii’. This study confirms the use of generic haemoplasma qPCRs to successfully amplify haemoplasma DNA from fixed, stained and archived blood smears from the early 1970s and provides molecular confirmation of the existence of a novel haemoplasma species in an owl monkey, for which the name ‘*Candidatus* Mycoplasma aoti’ sp. nov. is proposed.

## 1 Introduction

Haemoplasmas is the trivial name given to a group of uncultivated, erythrocyte parasitizing bacteria of the genus *Mycoplasma* within the Mollicutes class that can result in infectious anaemia (Neimark et al., 2001). They were reclassified as members of the genus *Mycoplasma* from the genera *Eperythrozoon* and *Haemobartonella* based on 16S rRNA gene phylogeny and have been previously identified using molecular diagnostics in the blood of mammals such as squirrel monkeys, cats, dogs, rodents, pigs, cattle, and sheep (Foley and Pedersen, 2001; Neimark et al., 2001, 2002, 2004; Sykes et al., 2005; Willi et al., 2005).

Historically, the diagnosis of haemoplasma infection relied on cytological examination of thin blood films, but this method lacks both sensitivity and specificity (Westfall et al., 2001; Tasker et al., 2003). Diagnosis of haemoplasma infection now relies on molecular methods, and a number of quantitative real-time PCR (qPCR) assays now exist for haemoplasmas (Willi et al., 2006; Peters et al., 2008b; Tasker et al., 2010).

In the late 1960s an owl monkey (*Aotus trivirgatus*) was imported into the United Kingdom from Colombia, and was splenectomised and experimentally infected with the protozoal parasite *Plasmodium falciparum*. Whilst observing the course of the infection, erythrocyte associated *‘Eperythrozoon*’-like bodies were described following microscopic examination of Giemsa-stained blood films and electron microscopy (Peters et al., 1974). Here we describe the use of a human glyceraldehyde-3-phosphate dehydrogenase (GAPDH) gene qPCR and generic haemoplasma qPCRs to confirm the presence of both amplifiable host DNA and haemoplasma DNA in archived smears. Partial 16S rRNA and ribonuclease P ribosomal (*rnpB*) gene amplification, cloning and sequencing was then used to provide phylogenetic data and distinguish the organism from existing named species.

## 2 Materials and methods

Three Giemsa-stained blood smears from this owl monkey had been archived and were available for analysis in the current study. The blood smears were examined by light microscopy. DNA was then extracted from each individual slide using the NucleoSpin^®^ Blood kit (Macherey-Nagel, ABgene, Epson, UK) according to a method described by Tasker et al. (2010). DNA from each extraction was subjected to qPCR utilising primers for the highly conserved human GAPDH gene as an internal DNA amplification control and qPCRs to screen for haemoplasma infections; generic haemoplasma haemominutum group (HM group; 139 bp amplicon) and haemofelis group (HF group; 110 bp amplicon) 16S rRNA gene qPCRs, as previously described (Tasker et al., 2010), all in duplicate. Positive (DNA extracted from *Mycoplasma haemofelis* or “*Candidatus* Mycoplasma haemominutum” infected cat blood samples and from a human blood smear) and negative (water) control reactions were included in each assay. The owl monkey GAPDH gene sequence was not available for analysis, however, this protein is highly conserved in mammals and analysis of the gene from the Rhesus Macaque (*Macaca mullata*; NM_001195426) indicated 100% identity at the primer binding sites and a single base pair mis-match in the probe binding site of the human GAPDH qPCR, suggesting that this assay would be able to detect owl monkey DNA. Amplification of an approximately 225 bp fragment of the *rnpB* gene was performed using primers 80F1, GAGGAAAGTCCRYGCTWGCAC and 290R1, TCCCYTACCRAAATTTRGGTTTCT (Birkenheuer et al., 2002) according to methods described by Peters et al. (2008a). Attempts to amplify larger fragments of the 16S rRNA gene were unsuccessful (data not shown). Haemoplasma 16S rRNA gene qPCR and *rnpB* gene amplicons from duplicate reactions were gel purified (NucleoSpin^®^ Extract II Kit; Macherey-Nagel), cloned into pCR^®^4-TOPO^®^ and transformed into *Escherichia coli* TOP10 cells (TOPO TA Cloning^®^ Kit, Invitrogen), then sequenced (DNA Sequencing & Services, www.dnaseq.co.uk).

BLASTn analysis was performed to compare the 16S rRNA and *rnpB* gene sequences to those in GenBank. A phylogenetic tree including existing haemoplasma species as well as selected non-haemoplasma *Mycoplasma* species, was constructed using Accelrys Gene v2.5 (Accelrys) for the *rnpB* gene using the neighbour-joining program from a distance matrix (Saitou and Nei, 1987), corrected for nucleotide substitutions by the Kimura two-parameter model (Kimura, 1980). The data set was resampled 1000 times to generate bootstrap percentages. Due to the limited haemoplasma 16S rRNA sequence data generated in the study, phylogenetic analysis was not performed for this gene.

## 3 Results and discussion

**Fig. 1:**
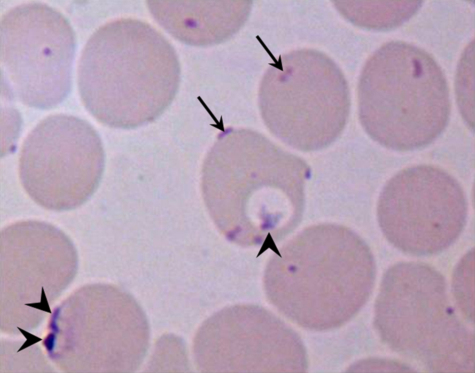
Light microscopy image of an archived Giemsa-stained blood smear from the owl monkey taken under high magnification (×1000, oil). Early trophozoites of *Plasmodium falciparum* (arrow heads) can be seen within the erythrocytes whilst haemoplasma bodies (arrows) can be seen on the cell membrane of the erythrocytes.

Light microscopic examination of the archived Giemsa-stained blood smears (Fig. 1) were in agreement with the original report, which stated that purple cocci and coccobacilli were seen on the erythrocyte surface (Peters et al., 1974). Morphologically distinct *Plasmodium* spp. early trophozoites were also seen within some erythrocytes, and some erythrocytes were seen to be parasitized by both malaria and haemoplasmas. In the original report, electron microscopy of infected blood had demonstrated organisms, most lying within indentations of the erythrocyte membrane, with features consistent with a haemotropic *Mycoplasma* spp. such as an absence of membrane bound organelles and the presence of strands of suspected DNA or ribosomes, as had been reported for other members of the *Eperythrozoon* and *Haemobartonella* genera (Tanaka et al., 1965).

**Fig. 2:**
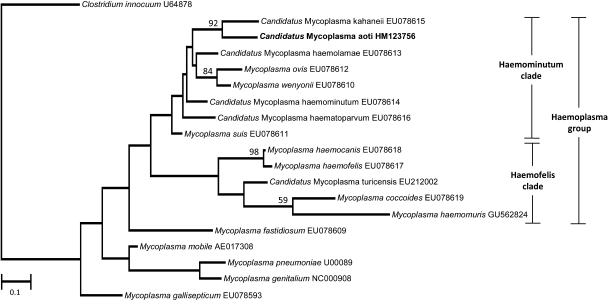
Phylogenetic analysis of partial ribonuclease P ribosomal gene sequences for the newly described haemoplasma species (shown in bold), other available haemoplasma species and selected non-haemoplasma *Mycoplasma* species. The phylogenetic tree was rooted to *Clostridium innocuum* (U64878). The tree was constructed by the neighbour-joining method. Evolutionary distances are to the scales shown. GenBank accession numbers are shown for all sequences.

The human GAPDH qPCR confirmed the extraction of amplifiable host DNA from the blood smears with threshold cycle (Ct) results of 30.3, 32.8, and 33.3. The presence of a haemoplasma species was confirmed in all three blood smear extractions using both the HM group (Ct values 26.3, 29.4, and 32.4) and HF group (Ct values 26.8, 29.3 and 32.4) qPCRs. Cross-reactivity between the HM and HF group assays can occur during the amplification of haemoplasma species, as reported elsewhere (Tasker et al., 2010), and this is thought to be dependent in part on the haemoplasma copy numbers present in the samples being amplified. Positive and negative PCR controls were appropriately positive and negative. Sequencing and BLASTn analysis of both HM and HF group qPCR amplicons (accession numbers HM123755 and HM123754) and the amplified fragment of the *rnpB* gene (HM123756), confirmed that the owl monkey haemoplasma belonged to the haemominutum clade. Analysis of the HM and HF group qPCR 16S rRNA gene amplicons and the *rnpB* gene amplicon revealed highest identity (96.4% for the 16S rRNA gene, 82.4% for the *rnpB* gene) to ‘*Candidatus* Mycoplasma kahaneii’ (AF338269, EU078615), a member of the haemominutum clade of haemoplasmas. The *rnpB* gene phylogenetic tree (Fig. 2) confirmed that this novel haemoplasma was most closely related to, but distinct from, ‘*Candidatus* M. kahaneii’, lying in the haemominutum clade.

Unfortunately the complete 16S rRNA gene sequence for the owl monkey haemoplasma could not be generated in the current study despite several attempts. It is suspected that the haemoplasma DNA had undergone degradation during the archive period such that longer gene fragments could not be amplified. However, although previous authors have suggested that 16S rRNA gene based phylogeny can be useful to ascribe a proposed novel species to a genus; it may not provide species level differentiation as some distinct species can share identities of greater than 97% across the near-complete 16S rRNA gene (Drancourt et al., 2000). In the current study we were able to generate adequate *rnpB* gene sequence data for phylogeny studies. Phylogenetic analysis of the *rnpB* gene has been suggested for species level differentiation with interspecies identities of 80–85% compared to intraspecies identities of ≥97% within one genus (Yoon and Park, 2000). The haemominutum clade interspecies *rnpB* gene identities range from 79.8 to 88.8%, supporting our conclusion that the haemoplasma species from the owl monkey identified in this study is a distinct novel species. It is of interest that the haemoplasma species most closely related to this novel owl monkey species is ‘*Candidatus* M. kahaneii’, which was reported in squirrel monkeys originating from the same geographical region (Neimark et al., 2002) as the owl monkey in this report. This may indicate ‘recent’ divergence from a common haemoplasma ancestor with a primate host.

It is suspected that splenectomy of the owl monkey compromised its immune response resulting in recrudescence of the haemoplasma infection. Apparent recrudescence of haemoplasma infection following splenectomy has also been reported in other host species, including squirrel monkeys, mice and dogs used in experimental procedures (Peters, 1965; Neimark et al., 2002; Kemming et al., 2004).

This study provides molecular confirmation of the existence of a novel haemoplasma species in an owl monkey, previously only suspected microscopically. The name ‘*Candidatus* Mycoplasma aoti’ is proposed for this novel haemoplasma species. Additionally, this report confirms the use of generic haemoplasma qPCRs to successfully amplify haemoplasma DNA from fixed, stained and archived blood smears that were over 40 years old.
